# Data-Driven Pressure Drop Prediction in Corrugated Pipe Extrusion: A Production-Based Power Law Approach

**DOI:** 10.3390/polym18131601

**Published:** 2026-06-27

**Authors:** Marco Cinquini, Giorgio Ramorino, Anna Gobetti

**Affiliations:** Mechanical and Industrial Engineering Department, University of Brescia, Via Branze 38, 25123 Brescia, Italy; giorgio.ramorino@unibs.it (G.R.); anna.gobetti@unibs.it (A.G.)

**Keywords:** extrusion, die design, pressure drop, power law, corrugated pipes, production data, predictive modeling

## Abstract

While data fitting is extensively used in polymer processing to extract fundamental rheological properties, its application for direct macroscopic geometric transfer between complex operational dies remains largely unexplored. Optimizing extrusion dies for corrugated plastic pipes traditionally requires time-consuming offline laboratory rheology, creating a major development bottleneck when dealing with proprietary, undocumented blends. To address this gap, this study introduces a novel, data-driven protocol for predicting the die pressure drop that eliminates the need for independent laboratory rheometry. Unlike traditional in situ methods that seek pure material properties, our approach back-calculates lumped, effective Power Law parameters directly from macroscopic pressure drops of existing converging dies. This uniquely embeds both material and geometric flow characteristics under actual processing conditions. Experimental validation demonstrates that this workflow, supported by an iterative refinement strategy, yields prediction errors typically within 10%. Ultimately, this lightweight computational tool provides engineers with a rapid-iteration framework to significantly accelerate early-stage die design.

## 1. Introduction

Corrugated plastic pipes are indispensable across diverse industry sectors, from infrastructure to healthcare, due to their robustness and adaptability. They serve as vital conduits for applications including protecting electrical cables, managing water and drainage lines, facilitating fluid and gas transport, and safeguarding wiring harnesses in the automotive and medical industries [1,2]. To meet the escalating global demand for these components, optimizing the manufacturing process is essential. A critical factor in this optimization is the accurate prediction of the pressure drop within extrusion dies. This parameter directly influences achievable mass flow rates, thereby impacting both production efficiency and the overall quality of the manufactured pipes [3].

In the current state of the research field, achieving accurate pressure drop predictions for optimal die design remains a challenge. While Computational Fluid Dynamics (CFD) offers detailed insights, and recent state-of-the-art approaches have successfully integrated CFD with high-performance computing [4] and machine learning algorithms for automated die design [5,6], these high-fidelity numerical simulations are computationally expensive. More importantly, they strictly require comprehensive rheological data—specifically, a full viscosity curve across a broad shear rate range. However, in industrial practice, pipe manufacturers frequently rely on specific, proprietary blends of raw resins, recycled materials, and additives to optimize production costs [7,8,9,10]. Because material suppliers typically only provide single-point data like the Melt Flow Index (MFI)—which oversimplifies the complex shear rate distribution within a die—designers are left without the comprehensive viscosity curves required to accurately model these unique compositions. Consequently, a die geometry optimized for standard parameters will perform unpredictably with a pipe manufacturer’s specific blend. Furthermore, while analytical models based on constitutive equations, such as the Power Law model [11,12], have historically been fundamental to pressure drop prediction, the computational overhead and the complexity of applying specific empirical correction factors can hinder rapid implementation during the initial design phase [13].

To overcome the limitations of offline characterization, the concept of utilizing extrusion process data to infer rheological properties—often termed in-line or in situ rheometry—has emerged as a recognized subfield in polymer processing [14]. Researchers have long noted that when offline characterization is unreliable or impossible, in-process methods offer a highly practical alternative [15]. For instance, several studies have successfully extracted fundamental viscosity and shear rate relationships by minimizing the difference between measured and calculated pressures in specific, controlled geometries, such as flat dies [16] or rectangular channels [17]. Other advanced approaches have employed inverse methods to estimate rheological laws directly from internal flow and temperature measurements [18].

However, a critical gap remains in the literature regarding industrial application. While data fitting techniques to determine rheological parameters have been extensively studied and successfully used for process control, these established in situ techniques primarily aim to reconstruct the fundamental, true viscosity curve of the material. Consequently, they tightly rely on simple, highly controlled geometries (e.g., slit or capillary channels) to act as on-board rheometers. They are not explicitly designed for macroscopic geometric transfer—the engineering act of using overall pressure data from one highly complex, converging annular die to predict the performance of an entirely different die geometry. In the production of corrugated pipes, where geometries are intricate and materials are often undocumented proprietary blends, attempting to extract pure, fundamental rheological data is practically unfeasible. The unique contribution of this work lies in shifting the paradigm: rather than using data fitting to find absolute material properties, we identify a set of global, effective parameters that mathematically lump together the integrated macroscopic behavior of the specific die-blend combination under actual thermal and shear conditions. Ultimately, this approach aims to provide an accessible, high-speed computational tool that achieves prediction errors typically within 10%, allowing die designers to rapidly iterate new geometries tailored to a manufacturer’s specific material reality.

## 2. Materials and Methods

### 2.1. Theoretical Framework and Constitutive Equations

Estimating pressure drop within extrusion dies for corrugated plastic pipes is inherently complex due to the non-Newtonian nature of the polymer melt. Unlike Newtonian fluids, molten plastics exhibit shear-thinning behavior, where viscosity decreases significantly as the shear rate increases. This dependency complicates pressure drop predictions across the varying flow conditions characteristic of complex die geometries.

To model this behavior analytically, the Power Law model is widely adopted. While it possesses known limitations—specifically its inability to describe the Newtonian plateau and its physically unrealistic prediction of infinite viscosity at zero-shear rates (for n<1), its mathematical simplicity makes it a robust tool for engineering calculations within the relevant processing window. The model defines the relationship between shear stress (τ) and shear rate ($\dot{\gamma }$) as follows:

(1) $$\tau =C\cdot {\dot{\gamma }}^{n}$$

where

*C:* is the flow consistency index (a measure of the fluid’s resistance to flow) $\left[Pa\cdot s^{n}\right]$;

*n*: is the flow behavior index [dimensionless].

For Newtonian fluids, n=1, and the relationship simplifies to Newtonian behavior. For non-Newtonian fluids, n<1, indicating shear-thinning behavior.

The relationship between dynamic viscosity (η) and shear rate for non-Newtonian fluids described by the Power Law model can be expressed as:

(2) $$\eta =\frac{\tau }{\dot{\gamma }}=\frac{C\cdot {\dot{\gamma }}^{n}}{\dot{\gamma }}=\frac{{\left(\frac{\dot{\gamma }}{C^{-\frac{1}{n}}}\right)}^{n}}{\dot{\gamma }}=\frac{{\left(\frac{\dot{\gamma }}{\phi }\right)}^{\frac{1}{m}}}{\dot{\gamma }}=\phi^{-\frac{1}{m}}\cdot {\dot{\gamma }}^{\frac{1}{m}-1}$$

where $\phi =C^{-1/n}$ and m=1/n.

Rearranging Equation (2) yields:

(3) $$\log\eta =\left(\frac{1}{m}-1\right)\log\dot{\gamma }-\frac{1}{m}\log\phi$$

As shown in Equation (3) the Power Law model dictates a linear relationship between the logarithms of dynamic viscosity and shear rate, enabling the straightforward extraction of model parameters from experimental data. Crucially, for a fluid that perfectly adheres to the Power Law model, m and ϕ would be constant material properties across all shear rates at a given temperature.

**Figure 1:**
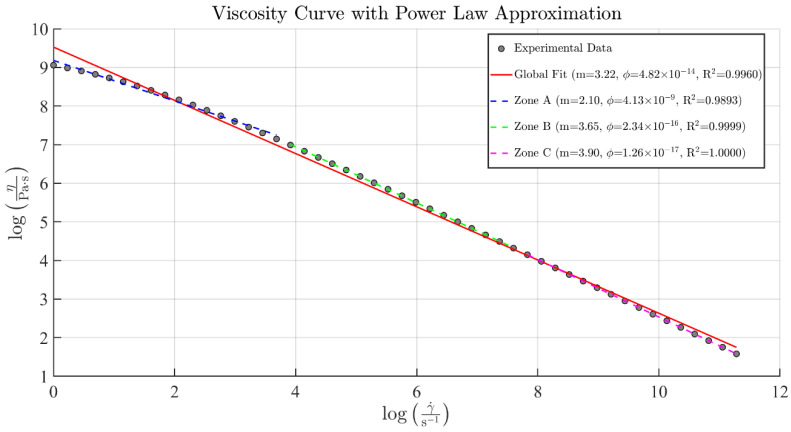
Viscosity curve of Polypropylene PP Borealis BB125MO as a function of shear rate, presenting experimental data, an overall (global) Power Law approximation, and three distinct Power Law regions (Zone A, B, and C). The experimental data, collected at 200 °C using a rheometer, are shown as black circles. The global fit is represented by a solid red line, while the zone-specific fits are shown as dashed lines in blue (Zone A), green (Zone B), and magenta (Zone C). Each fit is accompanied by its corresponding Power Law index (m), consistency index (ϕ), and coefficient of determination ($R^{2}$) in the legend. The generally higher $R^{2}$ values for the zone-specific fits, (with the exception of Zone A), compared to the global approximation, illustrate the enhanced accuracy gained by adapting Power Law parameters to local shear rate environments, a strategy employed in this work to model complex die flows. Shear rate ($\dot{\gamma }$) and viscosity (η) are presented on a natural logarithmic scale for linearized representation of the Power Law model.

However, in reality, experimental viscosity data for polymer melts, when plotted on a *log-log* scale, often present a non-linear curve, as illustrated in Figure 1. This non-linearity arises because polymers deviate from ideal Power Law behavior, particularly at very low and very high shear rates where Newtonian plateaus typically occur (corresponding to the zero-shear and infinite-shear rate viscosities, respectively). Therefore, a single set of constant Power Law parameters may not accurately represent the fluid’s behavior across a wide range of shear rates.

From an engineering perspective, maintaining predictive accuracy across the broad shear rate ranges found in complex extrusion dies requires a pragmatic approach. Rather than using a single, generalized set of Power Law parameters (m and ϕ) for all conditions, we determine the best-fit values specifically for the shear rate range experienced by a given die. Because complex die geometries—especially those with converging sections—induce a wide range of local shear rates, we first discretize the die into numerous small annular straight segments to identify the representative wall shear rates for a given flow condition. We then determine the specific $\bar{m}$ and $\bar{\phi }$ values that are uniquely tailored to the shear rate distribution characteristic of that die’s response. This approach ensures that the Power Law parameters accurately represent the material’s behavior within the actual operating window of the die. As shown in Figure 1, distinct effective parameters are necessary to capture the material response accurately across different shear rate regimes.

The Power Law model allows for the development of analytical solutions for various flow geometries, including those relevant to extrusion die design [19]. The volumetric flow rate ($\dot{V}$) [m^3^/s] through an annulus can be expressed as:

(4) $$\dot{V}=K\cdot \phi \cdot {\Delta p}^{m}$$

where K is the die conductance, a geometric factor influencing flow rate, and Δp [Pa] is the pressure drop.

For a straight concentric annulus, the die conductance (K) is given by:

(5) $$K=\frac{\pi DH^{m+2}}{2^{m+1}\left(m+2\right)}{\left(\frac{1}{L}\right)}^{m}$$

where

- D: Mean diameter of the annulus $\left(OD+ID\right)/2$ [m];
- H: Annulus thickness $\left(OD-ID\right)/2$ [m];
- L: Annulus length [m].

Also, in [19], an expression for the shear rate at the wall (${\dot{\gamma }}_{W}$ in [1/s]) for a concentric straight annulus is proposed:

(6) $${\dot{\gamma }}_{W}=\frac{2(m+2)\dot{V}}{\pi DH^{2}}$$

It is important to note that the relationships presented in Equations (5) and (6) are derived under the following assumptions:

1. Isothermal flow.
2. Incompressible flow.
3. No-slip boundary condition.
4. Steady-state flow.
5. Laminar flow.
6. Neglect of inlet and outlet effects.

While these analytical assumptions represent a simplified view of complex non-Newtonian flow, their physical validity and range of applicability within this data-driven framework are justified as follows:

1. Isothermal Flow: True isothermal behavior is rarely achieved in converging dies due to viscous dissipation (shear heating). However, because our methodology relies on macroscopic pressure drops measured directly from the operational die, the empirically back-calculated parameters ($\bar{m}$, $\bar{\phi }$) intrinsically capture and average out these thermal gradients across the evaluated operating window.
2. Incompressible Flow: Polymeric melts are widely modeled as incompressible under the relatively low-pressure gradients typical of the die-forming phase in extrusion. (see [20] for p-v-T diagrams).
3. No-Slip Boundary Condition: While certain polymers (e.g., HDPE) can exhibit wall slip at elevated shear stresses, standard processing conditions for corrugated pipes are maintained below the critical shear stress threshold for gross melt fracture. Furthermore, any minor, steady-state microscopic slip is inherently lumped into the global effective parameters during the back-calculation process.
4. Steady-State Flow: Maintaining a stable extrusion process is essential for ensuring the quality of products.
5. Laminar Flow: Due to the extremely high viscosity of polymer melts, the Reynolds number in pipe extrusion is typically in the creeping flow regime (Re≪1), rendering the assumption of strictly laminar flow physically robust.
6. Inlet and Outlet Effects: In this proposed workflow, converging geometries and inlet pressure drops are not ignored; rather, they are mathematically incorporated into the overall equivalent die conductance ($K_{eq}$) derived from the reference experimental data. The empirical extraction ensures that these geometry-induced pressure losses are reflected in the global effective parameters.

**Figure 2:**
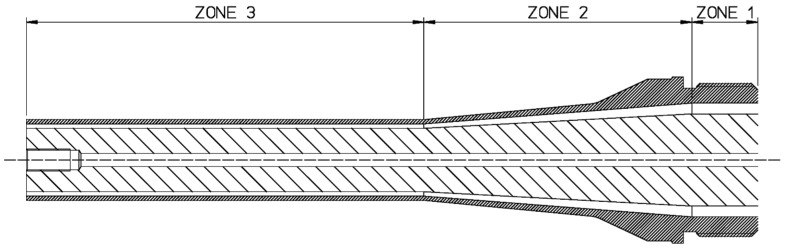
Schematic drawing of an extrusion die for single-wall corrugated pipe production, sectioned along the extrusion axis.

Figure 2 shows a typical extrusion die for the production of single-wall round corrugated plastic pipes. A typical extrusion die for corrugated plastic pipes consists of three main zones: a straight inlet section (Zone 1), a converging section (Zone 2), and a straight outlet section (Zone 3). In some cases, a further constriction section can be added. As previously established, the non-linear viscosity curve means that the true effective Power Law parameters would differ in each segment due to varying local shear rates. However, determining these numerous local parameters is impractical for a predictive model.

Therefore, we model the system by characterizing the entire die’s integrated flow response with a single set of overall effective parameters, $\bar{m}$ and $\bar{\phi }$. These parameters represent the global flow behavior of the material within the specific die geometry across the intended operating range.

Under this unified approach, the total pressure drop (Δp) is the sum of the pressure drops in each segment, all governed by the same $\bar{m}$ and $\bar{\phi }$. The die conductance for each segment (${\bar{K}}_{j}$) is calculated using the constant $\bar{m}$, making the total pressure drop:

(7) $$\Delta p={\left(\frac{\dot{V}}{{\bar{K}}_{1}\cdot \bar{\phi }}\right)}^{1/\bar{m}}+\sum_{s=1}^{t}\left[{\left(\frac{\dot{V}}{{\bar{K}}_{2(s)}\cdot \bar{\phi }}\right)}^{1/\bar{m}}\right]+{\left(\frac{\dot{V}}{{\bar{K}}_{3}\cdot \bar{\phi }}\right)}^{1/\bar{m}}$$

where the subscript j in ${\bar{K}}_{j}$ identifies the die zone and the second zone (j=2) was divided in t subparts.

The challenge, addressed in the following section, is to determine the two unknown effective parameters, $\bar{m}$ and $\bar{\phi }$, using experimental data. Accurate material rheology is the cornerstone of any predictive flow model. We are now at a juncture identical to that of a CFD simulation: the model is defined, but it requires the input of a viscosity curve to produce results.

While CFD excels at calculating detailed local shear distributions, its industrial utility is often bottlenecked by the requirement for comprehensive, precise viscosity data—which is frequently unavailable, proprietary, or too costly to obtain for every new material blend. Our methodology offers a pragmatic bypass to this hurdle. Instead of relying on laboratory rheometry, we leverage the existing production equipment as a rheometer, using overall pressure drop measurements from operational setups to empirically determine the global effective parameters.

This approach turns existing production data into actionable rheological insight, providing an efficient means for pressure drop estimation when full laboratory characterization is impractical. It serves as a complementary engineering tool, enabling rapid design iterations by deriving the necessary $\bar{m}$ and $\bar{\phi }$ parameters directly from the actual processing environment.

### 2.2. Extrapolation of $\bar{m}$ and $\bar{\phi }$ from Existing Pressure Drop Data

Pressure drop data from existing extrusion dies are often available to designers. Alternatively, this data can be gathered from operational feedback from existing dies. Many extruders are equipped with a melt sensor [21] that detects pressure and temperature at the extruder’s end, allowing for measurement of the combined pressure drop of the extrusion head (the main body that distributes the molten plastic) and the die (the interchangeable tooling that forms the final shape). By detaching the die and performing a test at the same flow rate, the pressure drop of the head alone can be found. The pressure drop due to the die is then isolated by subtraction:

(8) $$\Delta p_{die}=\Delta p_{die+head}-\Delta p_{head}$$

To determine the two unknown effective parameters, $\bar{m}$ and $\bar{\phi }$, in the pressure drop model, Equation (7), we use two experimental data points from an existing die. Let (${\dot{V}}_{A}$, $\Delta p_{A}$) and (${\dot{V}}_{B}$, $\Delta p_{B}$) be two pairs of volumetric flow rate and the corresponding measured overall die pressure drop. Applying these two conditions to the model yields the following system of equations:

(9) $$\left\{\begin{array}{ll} \Delta p_{A} & ={\left(\frac{{\dot{V}}_{A}}{{\bar{K}}_{1}\cdot \bar{\phi }}\right)}^{1/\bar{m}}+\sum_{s=1}^{t}{\left(\frac{{\dot{V}}_{A}}{{\bar{K}}_{2(s)}\cdot \bar{\phi }}\right)}^{1/\bar{m}}+{\left(\frac{{\dot{V}}_{A}}{{\bar{K}}_{3}\cdot \bar{\phi }}\right)}^{1/\bar{m}}=\\ & ={\left(\frac{{\dot{V}}_{A}}{\bar{\phi }}\right)}^{1/\bar{m}}\cdot \left[{\left(\frac{1}{{\bar{K}}_{1}}\right)}^{1/\bar{m}}+\sum_{s=1}^{t}{\left(\frac{1}{{\bar{K}}_{2(s)}}\right)}^{1/\bar{m}}+{\left(\frac{1}{{\bar{K}}_{3}}\right)}^{1/\bar{m}}\right]\\ \Delta p_{B} & ={\left(\frac{{\dot{V}}_{B}}{{\bar{K}}_{1}\cdot \bar{\phi }}\right)}^{1/\bar{m}}+\sum_{s=1}^{t}{\left(\frac{{\dot{V}}_{B}}{{\bar{K}}_{2(s)}\cdot \bar{\phi }}\right)}^{1/\bar{m}}+{\left(\frac{{\dot{V}}_{B}}{{\bar{K}}_{3}\cdot \bar{\phi }}\right)}^{1/\bar{m}}=\\ & ={\left(\frac{{\dot{V}}_{B}}{\bar{\phi }}\right)}^{1/\bar{m}}\cdot \left[{\left(\frac{1}{{\bar{K}}_{1}}\right)}^{1/\bar{m}}+\sum_{s=1}^{t}{\left(\frac{1}{{\bar{K}}_{2(s)}}\right)}^{1/\bar{m}}+{\left(\frac{1}{{\bar{K}}_{3}}\right)}^{1/\bar{m}}\right] \end{array}\right.$$

Since the geometric term in the square brackets represents the fixed geometry of the die and is therefore identical for both operating conditions (A and B), dividing the two equations allows us to cancel out the geometric factors and rearrange the terms to yield a direct solution for $\bar{m}$:

(10) $$\bar{m}=\frac{ln\left[{\dot{V}}_{A}/{\dot{V}}_{B}\right]}{ln\left[\Delta p_{A}/\Delta p_{B}\right]}$$

The deliberate choice to utilize exactly two operating conditions (*A* and *B*) to calibrate the model is rooted in both mathematical necessity and industrial practicality. Mathematically, deriving the two unknown effective parameters ($\bar{m}$, $\bar{\phi }$) requires a minimum of two independent data points to yield a deterministic algebraic solution. From an industrial standpoint, this drastically minimizes the data-gathering burden: a machine operator only needs to record the pressure drop at two distinct extruder speeds rather than conducting a comprehensive, multi-point rheological campaign. Furthermore, while a multi-point least-squares regression could generate a single set of parameters over a broader range, this global fit inevitably sacrifices local accuracy due to the non-linear nature of the polymer’s actual viscosity curve. Therefore, by purposefully selecting two calibration points that closely bracket the expected shear rate of the new in-design die, the protocol extracts a highly optimized local approximation. As demonstrated later in Section 3.3, this localized parameter identification yields superior predictive accuracy for the specific operational window of interest.

By substituting the calculated value of $\bar{m}$ back into either equation in (9), the value of $\bar{\phi }$ can be determined. With both $\bar{m}$ and $\bar{\phi }$ known, the pressure drop ($\Delta p_{d}$) for a new, in-design die with a geometry (${\bar{K}}_{d,j}$) and target volume flow rate (${\dot{V}}_{d}$) can be predicted, under the strict condition that it processes the exact same material or proprietary blend:

(11) $$\Delta p_{d}={\left(\frac{{\dot{V}}_{d}}{\bar{\phi }}\right)}^{1/\bar{m}}\cdot \left[{\left(\frac{1}{{\bar{K}}_{d,1}}\right)}^{1/\bar{m}}+\sum_{s=1}^{t}{\left(\frac{1}{{\bar{K}}_{d,2(s)}}\right)}^{1/\bar{m}}+{\left(\frac{1}{{\bar{K}}_{d,3}}\right)}^{1/\bar{m}}\right]$$

### 2.3. Validation via Similarity Condition

For this extrapolation to be valid, the new in-design die must process the same material, operate at the same temperature and within a similar shear rate range as the reference die. However, comparing the overall shear rate of two different, complex geometries is not straightforward. To address this, we introduce the mathematical construct of an *equivalent* straight annulus. This allows us to calculate a single, representative shear rate (${\dot{\gamma }}_{Weq}$) that characterizes the entire die’s integrated response for a specific flow condition. This metric enables a direct and meaningful comparison of the shear intensity experienced by the material across different geometries. We define an equivalent die conductance, $K_{eq}$, by equating the aggregate geometric term bracketed in Equation (9) to the standard definition of conductance for a straight annulus given in Equation (5):

(12) $$K_{eq}={\left[{\left(\frac{1}{{\bar{K}}_{1}}\right)}^{1/\bar{m}}+\sum_{s=1}^{t}{\left(\frac{1}{{\bar{K}}_{2(s)}}\right)}^{1/\bar{m}}+{\left(\frac{1}{{\bar{K}}_{3}}\right)}^{1/\bar{m}}\right]}^{-\bar{m}}=\frac{\pi D_{eq}H_{eq}^{\bar{m}+2}}{2^{\bar{m}+1}(\bar{m}+2)}{\left(\frac{1}{L_{eq}}\right)}^{\bar{m}}$$

This $K_{eq}$ can be mapped to the geometry of the equivalent straight annulus ($D_{eq}$, $H_{eq}$ and $L_{eq}$). We define $D_{eq}$ and $L_{eq}$ as consistent reference values (e.g., $D_{eq}=1/\pi$ and $L_{eq}=1$). Since these values are used consistently for both the reference and in-design dies, their specific magnitudes are not physically relevant and serve only to calculate a characteristic equivalent thickness, $H_{eq}$:

(13) $$H_{eq}={\left[K_{eq}\cdot 2^{\bar{m}+1}(\bar{m}+2)\right]}^{\frac{1}{\bar{m}+2}}$$

Using this equivalent geometry, we can calculate the equivalent shear rate at the wall for the two *i*-th experimental condition A and B on the reference die:

(14) $${\dot{\gamma }}_{Weq\;i}=\bar{\phi }\;\Delta p_{i}^{\bar{m}}\cdot {\left(\frac{H_{eq}}{2L_{eq}}\right)}^{\bar{m}}=\bar{\phi }\;\Delta p_{i}^{\bar{m}}\cdot {\left(\frac{H_{eq}}{2}\right)}^{\bar{m}}$$

These two shear rates define the valid operating range. The extrapolation is considered acceptable if the equivalent shear rate of the in-design die, ${\dot{\gamma }}_{Weq\;d}=\bar{\phi }\;\Delta p_{d}^{\bar{m}}\cdot {\left(\frac{H_{eq\;d}}{2}\right)}^{\bar{m}}$, falls within this range.

This *similarity condition* is expressed as:

(15) $${\dot{\gamma }}_{Weq\;A}\leq {\dot{\gamma }}_{Weq\;d}\leq {\dot{\gamma }}_{Weq\;B}$$

where the value for ${\dot{\gamma }}_{Weq\;d}$ is obtained using the geometric parameters of the new in-design die to calculate the equivalent thickness $H_{eq\;d}$.

### 2.4. Improving Accuracy with Iteration

It is important to note that the similarity condition is an empirical observation; it is an indicator of reliability, not a guarantee of accuracy. To improve the robustness of this approach, an iterative convergence method is strongly suggested:

1. Collect additional data: Obtain pressure drop data from the existing die at more operating conditions to expand the validated shear rate range.
2. Refine parameters: Use the expanded dataset to refine the estimates of $\bar{m}$ and $\bar{\phi }$ to narrow the shear rate range.
3. Re-evaluate: Check that the similarity condition is still met with the refined parameters and expanded range.
4. Iterate: Repeat the process until the predicted pressure drop converges to a stable value.

This iterative approach ensures predictions are based on a broader and more reliable dataset, continuously improving the model’s accuracy.

### 2.5. Data Collection

**Figure 3:**
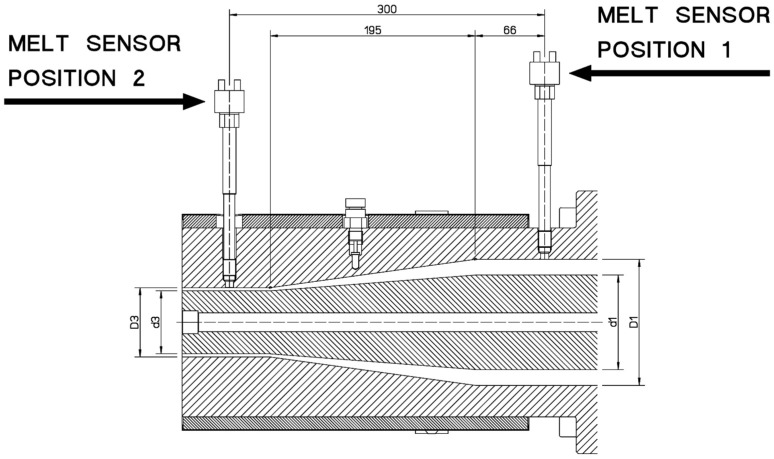
Schematic representation of the experimental setup for pressure drop measurements. Melt sensors are positioned at locations 1 and 2 along the die length to record pressure variations during the extrusion process. All dimensions are presented in mm.

To validate the proposed method, experiments were conducted using an industrial extrusion setup designed for processing PP and PE. The setup comprised:

- Extruder: An extruder with a screw diameter of 60 mm and a length-to-diameter (L/D) ratio of 30.
- Extrusion Head: A single-wall pipe extrusion head.
- Extrusion Dies: The setup used a single external die and three interchangeable internal dies to create three different annular gap geometries. The key dimensions, shown in the schematic in Figure 3, are:
  ○ $D_{1}$: Outer diameter at the die inlet (120 mm);
  ○ $d_{1}$: Inner diameter at the die inlet (90 mm);
  ○ $D_{3}$: Outer diameter at the die outlet (66 mm);
  ○ $d_{3}$: Inner diameter at the die outlet (variable).

The three interchangeable internal dies provided different outlet diameters ($d_{3}$ = 63, 60, and 57 mm) to achieve the target outlet gaps, $H_{3}$, of 1.5, 3.0, and 4.5 mm, respectively.

The external die was equipped with two melt sensors (pressure range: 0–50 MPa ± 0.5 MPa; temperature: type J thermocouple) connected to a PLC with a 1 Hz sampling rate. Heaters with feedback control maintained constant temperatures on the extrusion head and die surface.

Once the external and internal dies are coupled, the system is heated to the working temperature of 200 °C using the heating resistances. After the thermocouples installed in the extruder zones reach the desired temperature, several extrusion trials are conducted with the selected material.

The material is allowed to flow until a stable and uniform melt flow is achieved. Throughout the experiment, careful attention is paid to maintaining stable operating conditions, such as constant screw speed, consistent material feed rate, and uniform temperature distribution.

After stabilizing at the initial RPM, the extruder speed is increased to achieve the next mass flow rate. A waiting period of one minute is observed for each new RPM setting. Then, three samples of material are collected for one minute each and weighed on an analytical balance (to a precision of 0.01 g). The weight is recorded, and the system is monitored under steady-state conditions for 60 s to collect pressure and temperature data, yielding 60 continuous measurements per variable at the established 1 Hz sampling rate.

This process is repeated for each subsequent mass flow rate until the desired range is covered.

After completing the experiments with one internal extrusion die, the process is repeated with each of the other available internal extrusion dies to evaluate the effect of different outlet gaps.

The entire experiment is conducted with three different polymer blends: Polypropylene PP Borealis BB125MO (Borealis AG, Vienna, Austria), High-density polyethylene HDPE Hiplex^®^ HHM5502 (HIP-Petrohemija, Pančevo, Serbia), and Polypropylene PP Moplen EP440G (LyondellBasell, Rotterdam, The Netherlands).

A comprehensive evaluation of measurement errors, process variance, and standard uncertainty propagation is detailed in Appendix B.

Table 1 presents a representative sample of the experimental data collected for Polypropylene PP Borealis BB125MO (Borealis AG, Vienna, Austria). Complete datasets are available in Appendix A.

## 3. Results

This section presents the experimental validation of the proposed methodology. The analysis is structured in three parts. First, the self-consistency of the simplified Power Law model is verified for each individual die geometry. Second, a cross-validation test is performed to simulate a real-world design scenario, where data from a single reference die is used to predict the performance of two different in-design dies. Finally, the iterative refinement process is demonstrated as a tool for optimizing prediction accuracy.

### 3.1. Self-Consistency

To establish the baseline accuracy and self-consistency of the model, the effective parameters ($\bar{m}$, $\bar{\phi }$) were first derived from and applied to the same die geometry. This test verifies that the simplified Power Law model can accurately represent the flow behavior within a single, known configuration.

As shown in Table 2, Table 3 and Table 4, the model achieves excellent agreement with the experimental data. When applied to the same die from which its parameters were derived, the prediction error is consistently low, typically below 3%. This confirms that the simplified Power Law approach is a valid and accurate tool for characterizing the flow within a given die, providing a solid foundation for the cross-validation analysis.

### 3.2. Cross-Validation

The primary validation of the proposed methodology involves simulating a practical design task: predicting the performance of a new die using only data from a reference one. For this cross-validation test, the die with an outlet gap of $H_{3}$ = 1.5 mm was designated as the *existing* reference die. The parameters derived solely from this reference die’s experimental data were then used to predict the pressure drop for the two *in-design* dies with outlet gaps of $H_{3}$ = 3.0 mm and $H_{3}$ = 4.5 mm. The reference parameters and the corresponding valid shear rate range derived from the $H_{3}$ = 1.5 mm die are:

- $\bar{m}=3.3$;
- $\bar{\phi }=9.04\cdot 10^{-15}\;\frac{1}{Pa^{m}\cdot s}$;
- ${\dot{\gamma }}_{Weq\;A}=3.63\;s^{-1}$;
- ${\dot{\gamma }}_{Weq\;B}=22.22\;s^{-1}$.

Using these reference parameters, the predictive performance of the model for the new in-design dies is summarized in Table 5 (for the $H_{3}$ = 3.0 mm outlet gap) and Table 6 (for the $H_{3}$ = 4.5 mm outlet gap).

**Figure 4:**
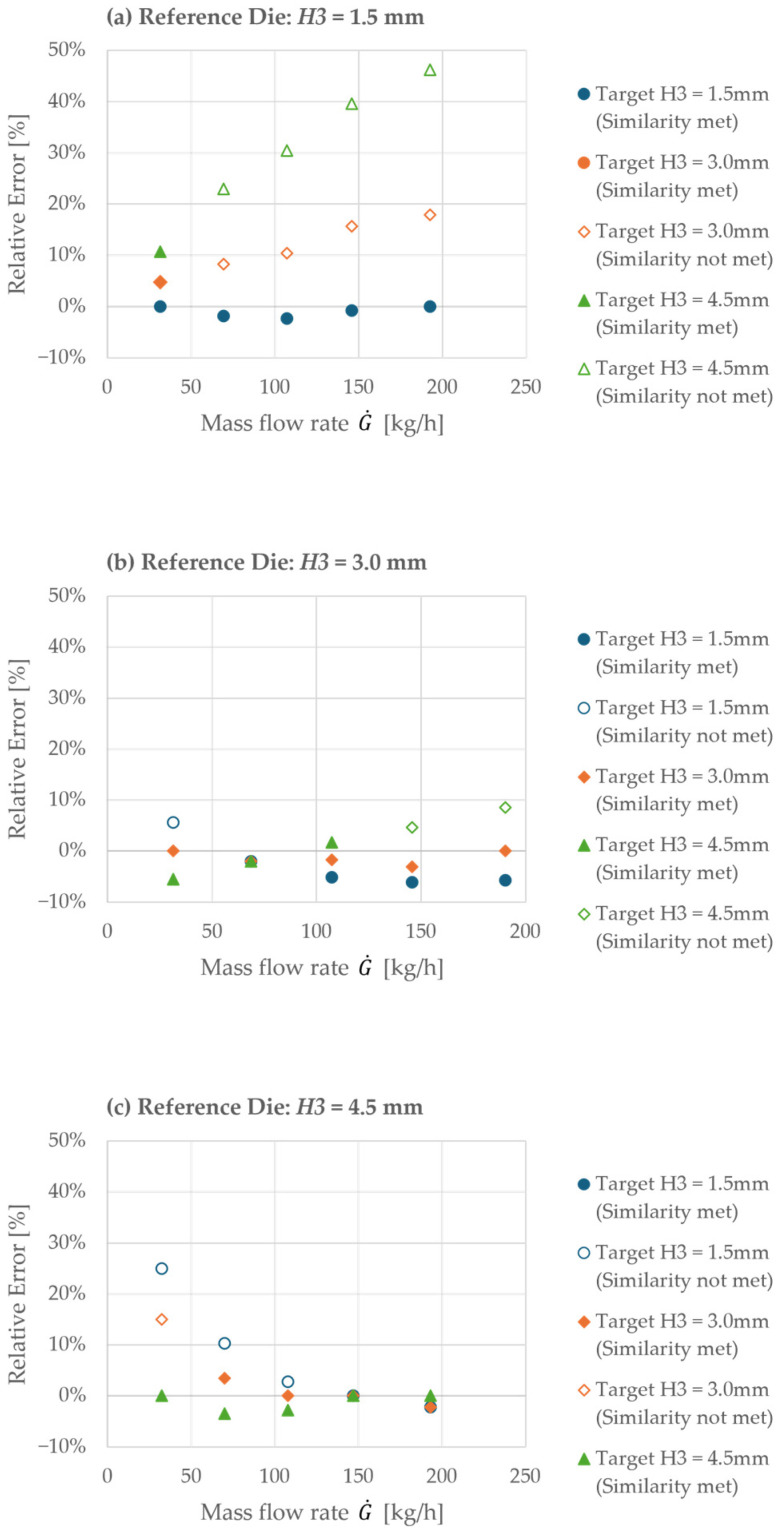
Relative prediction error versus mass flow rate ($\dot{G}$) for both self-consistency and cross-validation analyses. The multi-panel figure displays the predictive performance when utilizing: (**a**) reference die $H_{3}=1.5\;mm$; (**b**) reference die $H_{3}=3.0\;mm$; and (**c**) reference die $H_{3}=4.5\;mm$. Solid markers indicate predictions where the similarity condition is strictly satisfied, demonstrating high accuracy and stability (errors typically bounded within ±10%). Conversely, hollow markers indicate data points falling outside the validated equivalent shear rate range, which intrinsically lead to a severe divergence in the prediction error.

To comprehensively evaluate the robustness of the macroscopic geometric transfer, both a self-consistency check and a cross-validation analysis were conducted. In this phase, the effective Power Law parameters extracted from each reference die were used to predict the pressure drops of both their native geometry (self-consistency) and the other target geometries (cross-validation). To explicitly visualize the critical role of the similarity condition across all scenarios, the relative prediction errors are plotted against the mass flow rate ($\dot{G}$) in Figure 4. This multi-panel representation clearly delineates the operational boundary between a highly reliable geometric transfer and an unstable extrapolation zone, highlighting the necessity of operating within the validated equivalent shear rate range defined by the similarity condition.

### 3.3. Iterative Refinement

The methodology also provides a clear pathway for improving prediction accuracy by selecting a more appropriate reference data range. To illustrate this iterative refinement, consider the cross-prediction for the $H_{3}$ = 3.0 mm die at $\dot{G}$ = 145.80 kg/h, shown previously in Table 5 (and summarized here in Table 7).

This prediction was obtained within the valid shear rate range of $\left[3.63,22.22\right]\;s^{-1}$ defined by the similarity condition. The accuracy of the prediction can be further refined by selecting a narrower shear rate range; specifically, by using the experimental data from Table 2 at ${\dot{G}}_{A}=31.50\;kg/h$ and ${\dot{G}}_{B}=69.48\;kg/h$ as the reference values for the Power Law parameters, as shown in Table 8.

The values of $\bar{m}$, $\bar{\phi }$, ${\dot{\gamma }}_{Weq\;A}$ and ${\dot{\gamma }}_{Weq\;B}$ recalculated using these reference values are:

- $\bar{m}=3.0$;
- $\bar{\phi }=1.99\cdot 10^{-13}\;\frac{1}{Pa^{m}\cdot s}$;
- ${\dot{\gamma }}_{Weq\;A}=3.88\;s^{-1}$;
- ${\dot{\gamma }}_{Weq\;B}=8.56\;s^{-1}$.

With these refined parameters, we re-evaluate the prediction for our target mass flow rate of 145.80 kg/h.

As shown in Table 9, the new equivalent shear rate, ${\dot{\gamma }}_{Weq}=6.60\;s^{-1}$, comfortably satisfies the similarity condition within the narrower, more relevant range of $\left[3.88,8.56\right]\;s^{-1}$. Critically, the predicted pressure drop is once again 6.1 MPa. While the value itself has not changed, this result validates and strengthens the confidence in the original prediction, as it is now grounded in a reference dataset that more closely matches the target operating conditions.

**Figure 5:**
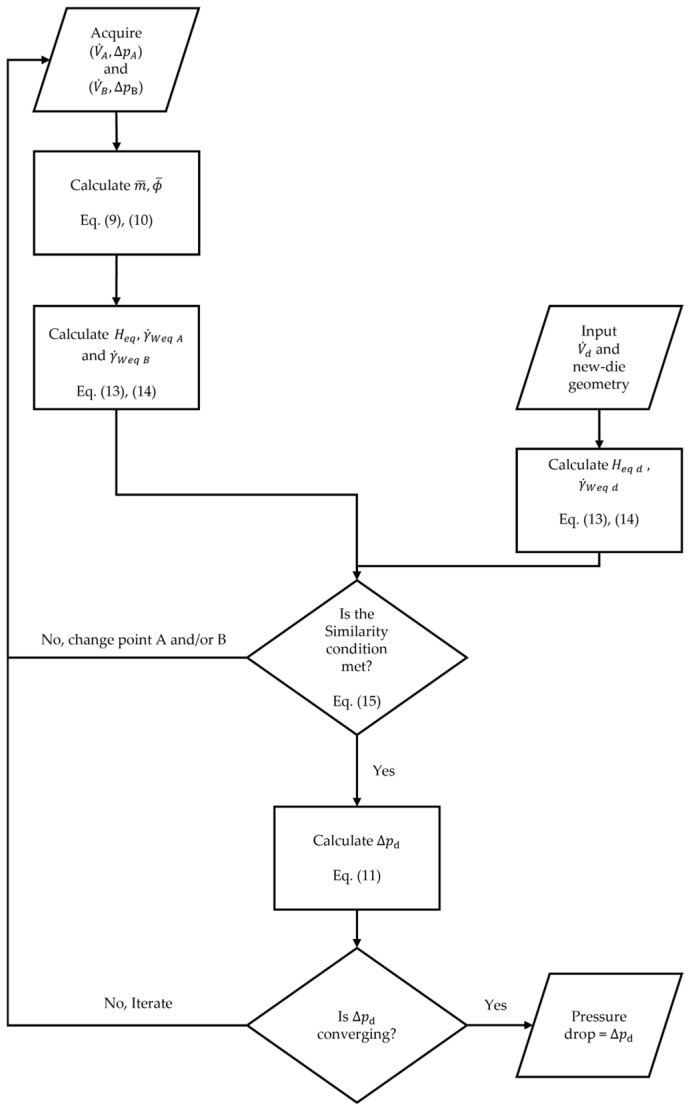
Algorithmic workflow of the proposed data-driven methodology for macroscopic geometric transfer and pressure drop prediction. The flowchart illustrates the extraction of the effective Power Law parameters ($\bar{m}$, $\bar{\phi }$) from a reference die, the validation via the similarity condition, and the iterative refinement strategy required to achieve convergence for a new in-design geometry.

To provide a clear and structured overview of the proposed methodology, the complete algorithmic workflow is summarized in Figure 5. This flowchart illustrates the step-by-step practical implementation of the theoretical framework discussed above. It delineates the process from the initial extraction of the effective Power Law parameters using macroscopic reference data, through the critical validation of the target equivalent shear rate via the similarity condition, and concludes with the final pressure drop prediction. Crucially, it integrates the iterative refinement loop, showing how the predictive accuracy for a new in-design die can be systematically optimized.

## 4. Discussion

This study aimed to develop a practical workflow for predicting the pressure drop inside extrusion dies for the production of corrugated plastic pipes. Rather than developing a new constitutive equation, this work successfully establishes an industrially oriented protocol for geometric transfer and parameter identification. This data-driven protocol utilizes pressure drop data collected from existing extrusion dies processing the same material and is further refined through an iterative refinement of the Power Law parameters. The results demonstrate that this approach yields pressure drop predictions with a relative error typically within 10%, strictly provided that the equivalent shear rate of the in-design geometry satisfies the empirical similarity condition established by the reference die. Furthermore, because the Power Law acts as a local secant approximation of the true non-linear viscosity curve, the predictive accuracy intrinsically increases as the target equivalent shear rate approaches either of the reference calibration points, or as the calibration interval itself is narrowed around the target. It is important to clarify that this methodology is intended as an offline, predictive engineering tool rather than a real-time, closed-loop optimization algorithm for active extrusion control. When implemented as a computational script, it generates rapid estimations using standard programming functions. By anchoring the analytical Power Law model directly to macroscopic production data, this methodology successfully bypasses the need for comprehensive laboratory rheometry, offering a mathematically validated, data-driven framework for initial die design evaluation.

Regarding the geometric scope of the validation, it is worth noting that the experimental campaign varied the die outlet gap from 1.5 mm to 4.5 mm. While the macro-architecture of the converging sections remained consistent, this factor-of-three variation represents a severe stress test within the context of corrugated pipe manufacturing. In industrial practice, die geometry fine-tuning for a specific pipe profile typically involves micro-adjustments in the order of tenths of a millimeter to control wall thickness distribution. Therefore, successfully predicting pressure drops across a multi-millimeter shift constitutes an extreme geometric variation for this tooling class. Furthermore, because the outlet gap acts as the narrowest flow constriction, it dictates the overwhelming majority of the total die pressure drop. Validating the macroscopic geometric transfer across such a broad range of the most sensitive flow-restrictive parameter robustly confirms the method’s efficacy for real-world die optimization.

While this study demonstrates the method’s utility, its primary limitations—namely the reliance on melt sensor accuracy and the specific set of geometries and materials tested—define clear directions for future research. Subsequent work should therefore focus on expanding the scope of this study by: (1) investigating a wider range of die geometries, including more complex configurations; (2) testing a broader spectrum of polymers with varying rheological properties; (3) exploring a wider range of operating conditions; and (4) developing more robust validation methods, potentially through comparisons with targeted CFD simulations.

## Figures and Tables

**Table 1:** Experimental data for Polypropylene PP Borealis BB125MO (Borealis AG, Vienna, Austria) collected at varying operating conditions. $H_{3}$ is the die outlet gap [mm], $\dot{G}$ is the mass flow rate [kg/h], N is the extruder screw speed [RPM], $p_{melt}$ is the melt pressure [MPa] (at the extruder exit), $p_{1}$ and $p_{2}$ are the pressures [MPa] at sensor locations one and two, $T_{1}$ and $T_{2}$ are the temperatures [°C] at sensor locations one and two, and $\Delta p_{EXP}$ is the pressure drop [MPa] calculated as $\left(p_{1}-p_{2}\right)$.

$H_{3}$ [mm]	$\dot{G}$ [kg/h]	N [RPM]	$p_{melt}$ [MPa]	$p_{1}$ [MPa]	$T_{1}$ [°C]	$p_{2}$ [MPa]	$T_{2}$ [°C]	$\Delta p_{EXP}$ [MPa]
1.5	31.50 ± 0.03	17	19.9 ± 0.5	14.8 ± 0.5	196.0 ± 2.5	6.4 ± 0.5	206.1 ± 2.5	8.4 ± 0.7
1.5	69.48 ± 0.06	39	25.9 ± 0.5	18.7 ± 0.5	196.4 ± 2.5	7.8 ± 0.5	206.6 ± 2.5	10.9 ± 0.7
1.5	107.22 ± 0.03	61	29.6 ± 0.5	21.0 ± 0.5	196.7 ± 2.5	8.5 ± 0.5	207.8 ± 2.5	12.5 ± 0.7
1.5	145.80 ± 0.01	84	32.2 ± 0.5	22.5 ± 0.5	196.9 ± 2.5	9.1 ± 0.5	209.6 ± 2.5	13.4 ± 0.7
1.5	192.6 ± 0.1	111	34.8 ± 0.5	24.0 ± 0.5	197.0 ± 2.5	9.5 ± 0.5	211.4 ± 2.5	14.5 ± 0.7
3.0	31.50 ± 0.05	17	10.6 ± 0.5	6.0 ± 0.5	193.6 ± 2.5	2.4 ± 0.5	201.4 ± 2.5	3.6 ± 0.7
3.0	68.52 ± 0.08	39	14.8 ± 0.5	7.8 ± 0.5	194.2 ± 2.5	2.9 ± 0.5	202.1 ± 2.5	4.9 ± 0.7
3.0	107.40 ± 0.09	61	17.5 ± 0.5	9.2 ± 0.5	194.7 ± 2.5	3.4 ± 0.5	202.9 ± 2.5	5.8 ± 0.7
3.0	145.80 ± 0.05	84	19.5 ± 0.5	10.1 ± 0.5	195.1 ± 2.5	3.6 ± 0.5	203.7 ± 2.5	6.5 ± 0.7
3.0	190.32 ± 0.15	111	21.1 ± 0.5	10.9 ± 0.5	195.6 ± 2.5	3.9 ± 0.5	205.0 ± 2.5	7.0 ± 0.7
4.5	32.28 ± 0.13	17	8.8 ± 0.5	3.9 ± 0.5	199.6 ± 2.5	1.9 ± 0.5	200.6 ± 2.5	2.0 ± 0.7
4.5	70.0 ± 0.3	39	11.3 ± 0.5	5.1 ± 0.5	199.9 ± 2.5	2.2 ± 0.5	201.0 ± 2.5	2.9 ± 0.7
4.5	107.9 ± 0.3	61	13.8 ± 0.5	6.0 ± 0.5	200.0 ± 2.5	2.4 ± 0.5	201.2 ± 2.5	3.6 ± 0.7
4.5	146.82 ± 0.16	84	15.8 ± 0.5	6.6 ± 0.5	200.1 ± 2.5	2.6 ± 0.5	201.4 ± 2.5	4.0 ± 0.7
4.5	193.14 ± 0.17	111	17.4 ± 0.5	7.2 ± 0.5	200.0 ± 2.5	2.7 ± 0.5	201.8 ± 2.5	4.5 ± 0.7

**Table 2:** Comparison between experimental and predicted pressure drop data for Polypropylene PP Borealis BB125MO (Borealis AG, Vienna, Austria) at varying mass flow rates ($\dot{G}$) for an outlet gap of $H_{3}=1.5\;mm$. Here, ${\dot{\gamma }}_{Weq}$ is the equivalent shear rate at the wall calculated as in (14), $\Delta p_{EXP}$ is the experimental pressure drop, and $\Delta p_{PL}$ is the pressure drop predicted using the Power Law model with $\bar{m}=3.3$ and $\bar{\phi }=9.04\cdot 10^{-15}\;1/\left(Pa^{m}\cdot s\right)$. Reference values for the Power Law parameters were derived from experimental data at ${\dot{G}}_{A}=31.50\;kg/h$ and ${\dot{G}}_{B}=192.60\;kg/h$. Note that all the equivalent shear rates at the wall in this table fall within the valid range for the Power Law model: $\left[3.63,22.22\right]\;s^{-1}$. For the specific standard deviations associated with the experimental mass flow rates ($\dot{G}$), please refer to the primary data in Table 1.

$\dot{G}$ [kg/h]	${\dot{\gamma }}_{Weq}$ [s^−1^]	$\Delta p_{EXP}\pm 0.7$ [MPa]	$\Delta p_{PL}$ [MPa]	Relative Error (%)
31.50	3.63	8.4	8.4	<0.01%
69.48	8.02	10.9	10.7	−1.83%
107.22	12.37	12.5	12.2	−2.40%
145.80	16.82	13.4	13.3	−0.75%
192.60	22.22	14.5	14.5	<0.01%

**Table 3:** Comparison between experimental and predicted pressure drop data for Polypropylene PP Borealis BB125MO (Borealis AG, Vienna, Austria) at varying mass flow rates ($\dot{G}$) for an outlet gap of $H_{3}=3.0\;mm$. Here, ${\dot{\gamma }}_{Weq}$ is the equivalent shear rate at the wall calculated as in (14), $\Delta p_{EXP}$ is the experimental pressure drop, and $\Delta p_{PL}$ is the pressure drop predicted using the Power Law model with $\bar{m}=2.7$ and $\bar{\phi }=6.83\cdot 10^{-12}\;1/\left(Pa^{m}\cdot s\right)$. Reference values for the Power Law parameters were derived from experimental data at ${\dot{G}}_{A}=31.50\;kg/h$ and ${\dot{G}}_{B}=190.32\;kg/h$. Note that all the equivalent shear rates at the wall in this table fall within the valid range for the Power Law model: $\left[1.53,9.25\right]\;s^{-1}$. For the specific standard deviations associated with the experimental mass flow rates ($\dot{G}$), please refer to the primary data in Table 1.

$\dot{G}$ [kg/h]	${\dot{\gamma }}_{Weq}$ [s^−1^]	$\Delta p_{EXP}\pm 0.7$ [MPa]	$\Delta p_{PL}$ [MPa]	Relative Error (%)
31.50	1.53	3.6	3.6	<0.01%
68.52	3.33	4.9	4.8	−2.04%
107.40	5.22	5.8	5.7	−1.72%
145.80	7.09	6.5	6.3	−3.08%
190.32	9.25	7.0	7.0	<0.01%

**Table 4:** Comparison between experimental and predicted pressure drop data for Polypropylene PP Borealis BB125MO (Borealis AG, Vienna, Austria) at varying mass flow rates ($\dot{G}$) for an outlet gap of $H_{3}=4.5\;mm$. Here, ${\dot{\gamma }}_{Weq}$ is the equivalent shear rate at the wall calculated as in (14), $\Delta p_{EXP}$ is the experimental pressure drop, and $\Delta p_{PL}$ is the pressure drop predicted using the Power Law model with $\bar{m}=2.2$ and $\bar{\phi }=1.50\cdot 10^{-9}\;1/\left(Pa^{m}\cdot s\right)$. Reference values for the Power Law parameters were derived from experimental data at ${\dot{G}}_{A}=32.28\;kg/h$ and ${\dot{G}}_{B}=193.14\;kg/h$. Note that all the equivalent shear rates at the wall in this table fall within the valid range for the Power Law model: $\left[1.04,6.21\right]\;s^{-1}$. For the specific standard deviations associated with the experimental mass flow rates ($\dot{G}$), please refer to the primary data in Table 1.

$\dot{G}$ [kg/h]	${\dot{\gamma }}_{Weq}$ [s^−1^]	$\Delta p_{EXP}\pm 0.7$ [MPa]	$\Delta p_{PL}$ [MPa]	Relative Error (%)
32.28	1.04	2.0	2.0	<0.01%
69.96	2.25	2.9	2.8	−3.45%
107.88	3.47	3.6	3.5	−2.78%
146.82	4.72	4.0	4.0	<0.01%
193.14	6.21	4.5	4.5	<0.01%

**Table 5:** Comparison between experimental and predicted pressure drop data for Polypropylene PP Borealis BB125MO (Borealis AG, Vienna, Austria) at varying mass flow rates ($\dot{G}$) for an outlet gap of $H_{3}=3.0\;mm$. Here, ${\dot{\gamma }}_{Weq}$ is the equivalent shear rate at the wall calculated as in (14), $\Delta p_{EXP}$ is the experimental pressure drop, and $\Delta p_{PL}$ is the pressure drop predicted using the Power Law model with $\bar{m}=3.3$ and $\bar{\phi }=9.04\cdot 10^{-15}\;1/\left(Pa^{m}\cdot s\right)$. Reference values for the Power Law parameters were derived from experimental data of Table 2 at ${\dot{G}}_{A}=31.50\;kg/h$ and ${\dot{G}}_{B}=192.60\;kg/h$. Note that the equivalent shear rates at the wall marked with * in this table do not fall within the valid range for the Power Law model $\left[3.63,22.22\right]\;s^{-1}$ and therefore do not satisfy the similarity condition. Consequently, the pressure drop predictions for these points may not be accurate and should be considered outside the validated range of applicability. For the specific standard deviations associated with the experimental mass flow rates ($\dot{G}$), please refer to the primary data in Table 1.

$\dot{G}$ [kg/h]	${\dot{\gamma }}_{Weq}$ [s^−1^]	$\Delta p_{EXP}\pm 0.7$ [MPa]	$\Delta p_{PL}$ [MPa]	Relative Error (%)
31.50	1.36 *	3.6	3.8	+5.56%
68.52	2.95 *	4.9	4.8	−2.04%
107.40	4.63	5.8	5.5	−5.17%
145.80	6.28	6.5	6.1	−6.15%
190.32	8.20	7.0	6.6	−5.71%

**Table 6:** Comparison between experimental and predicted pressure drop data for Polypropylene PP Borealis BB125MO (Borealis AG, Vienna, Austria) at varying mass flow rates ($\dot{G}$) for an outlet gap of $H_{3}=4.5\;mm$. Here, ${\dot{\gamma }}_{Weq}$ is the equivalent shear rate at the wall calculated as in (14), $\Delta p_{EXP}$ is the experimental pressure drop, and $\Delta p_{PL}$ is the pressure drop calculated using the Power Law model with $\bar{m}=3.3$ and $\bar{\phi }=9.04\cdot 10^{-15}\;1/\left(Pa^{m}\cdot s\right)$. Reference values for the Power Law parameters were derived from experimental data of Table 2 at ${\dot{G}}_{A}=31.50\;kg/h$ and ${\dot{G}}_{B}=192.60\;kg/h$. Note that the equivalent shear rates at the wall marked with * in this table do not fall within the valid range for the Power Law model $\left[3.63,22.22\right]\;s^{-1}$ and therefore do not satisfy the similarity condition. Consequently, the pressure drop predictions for these points may not be accurate and should be considered outside the validated range of applicability. For the specific standard deviations associated with the experimental mass flow rates ($\dot{G}$), please refer to the primary data in Table 1.

$\dot{G}$ [kg/h]	${\dot{\gamma }}_{Weq}$ [s^−1^]	$\Delta p_{EXP}\pm 0.7$ [MPa]	$\Delta p_{PL}$ [MPa]	Relative Error (%)
32.28	0.83 *	2.0	2.5	+25.00%
69.96	1.80 *	2.9	3.2	+10.34%
107.88	2.77 *	3.6	3.7	+2.78%
146.82	3.78	4.0	4.0	<0.01%
193.14	4.97	4.5	4.4	−2.22%

**Table 7:** Initial cross-prediction data for the die with an outlet gap of $H_{3}$ = 3.0 mm at a mass flow rate of 145.80 kg/h (extracted from Table 5).

$\dot{G}$ [kg/h]	${\dot{\gamma }}_{Weq}$ [s^−1^]	$\Delta p_{EXP}\pm 0.7$ [MPa]	$\Delta p_{PL}$ [MPa]
145.80	6.28	6.5	6.1

**Table 8:** Selected reference experimental data from the die with an outlet gap of $H_{3}$ = 1.5 mm used for the iterative refinement (extracted from Table 2).

$\dot{G}$ [kg/h]	${\dot{\gamma }}_{Weq}$ [s^−1^]	$\Delta p_{EXP}\pm 0.7$ [MPa]
31.50	3.63	8.4
69.48	8.02	10.9

**Table 9:** Comparison between experimental and iteratively refined predicted pressure drop data for Polypropylene PP Borealis BB125MO (Borealis AG, Vienna, Austria) at varying mass flow rates ($\dot{G}$) for an outlet gap of $H_{3}=3.0\;mm$. Here, ${\dot{\gamma }}_{Weq}$ is the equivalent shear rate at the wall calculated as in (14), $\Delta p_{EXP}$ is the experimental pressure drop, and $\Delta p_{PL}$ is the pressure drop predicted using the Power Law model with $\bar{m}=3.0$ and $\bar{\phi }=1.99\cdot 10^{-13}\;1/\left(Pa^{m}\cdot s\right)$. Reference values for the Power Law parameters were derived from experimental data of Table 2 at ${\dot{G}}_{A}=31.50\;kg/h$ and ${\dot{G}}_{B}=69.48\;kg/h$. Note that the equivalent shear rates at the wall marked with * in this table do not fall within the valid range for the Power Law model $\left[3.88,8.56\right]\;s^{-1}$ and therefore do not satisfy the similarity condition. Consequently, the pressure drop predictions for these points may not be accurate and should be considered outside the validated range of applicability. For the specific standard deviations associated with the experimental mass flow rates ($\dot{G}$), please refer to the primary data in Table 1.

$\dot{G}$ [kg/h]	${\dot{\gamma }}_{Weq}$ [s^−1^]	$\Delta p_{EXP}\pm 0.7$ [MPa]	$\Delta p_{PL}$ [MPa]	Relative Error (%)
31.50	1.43 *	3.6	3.7	+2.78%
68.52	3.10 *	4.9	4.7	−4.08%
107.40	4.86	5.8	5.5	−5.17%
145.80	6.60	6.5	6.1	−6.15%
190.32	8.61 *	7.0	6.6	−5.71%

## Data Availability

The original contributions presented in this study are included in the article. Further inquiries can be directed to the corresponding author.

## Appendix A

### Appendix A.1. Polypropylene PP Borealis BB125MO (Borealis AG, Vienna, Austria)

This section details the application of the pressure drop prediction methodology to Polypropylene PP Borealis BB125MO (Borealis AG, Vienna, Austria). Table A1 summarizes the self-consistency and cross-validation predictions. The results reinforce the main findings of this study: predictions are highly accurate when the similarity condition is met, and the error increases significantly when it is not, validating the condition as a reliable diagnostic tool.

**Table A1:** Relative error of pressure drop predictions for different Power Law model parameters at varying mass flow rates ($\dot{G}$) and outlet gap $H_{3}$ for Polypropylene PP Borealis BB125MO (Borealis AG, Vienna, Austria). The table displays the relative error (% with sign) between the experimental pressure drop and the pressure drop predicted by the Power Law model for various combinations of $\bar{m}$ and $\bar{\phi }$. Results located on the main diagonal represent self-consistency cases where the values of $\bar{m}$ and $\bar{\phi }$ used to predict the pressure drop were derived from the same die geometry. Conversely, values off the main diagonal represent cross-validated results, as the parameters were calculated using data from a different die. Values marked with an asterisk (*) indicate that the prediction does not meet the similarity condition and thus falls outside the model’s validated range of applicability. For the specific standard deviations associated with the experimental mass flow rates ($\dot{G}$), please refer to the primary data in Table 1.

In-Design Die $H_{3}$ [mm]	$\dot{G}$ [kg/h]	Relative Error (%)		
		Reference Die $H_{3}$ = 1.5 mm, $\bar{m}=3.3$ $\bar{\phi }=9.04\cdot 10^{-15}$ $\left[1/\left(Pa^{m}\cdot s\right)\right]$	Reference Die $H_{3}$ = 3.0 mm, $\bar{m}=2.7$ $\bar{\phi }=6.83\cdot 10^{-12}$ $\left[1/\left(Pa^{m}\cdot s\right)\right]$	Reference Die $H_{3}$ = 4.5 mm, $\bar{m}=2.2$ $\bar{\phi }=1.50\cdot 10^{-9}$ $\left[1/\left(Pa^{m}\cdot s\right)\right]$
1.5	31.5	<0.01%	4.76%	10.71%
	69.48	−1.83%	8.26% *	22.94% *
	107.22	−2.40%	10.40% *	30.40% *
	145.80	−0.75%	15.67% *	39.55% *
	192.60	<0.01%	17.93% *	46.21% *
3.0	31.50	5.56% *	<0.01%	−5.56%
	68.52	−2.04% *	−2.04%	−2.04%
	107.40	−5.17%	−1.72%	1.72%
	145.80	−6.15%	−3.08%	4.62% *
	190.32	−5.71%	<0.01%	8.57% *
4.5	32.28	25.00% *	15.00% *	<0.01%
	69.96	10.34% *	3.45%	−3.45%
	107.88	2.78% *	<0.01%	−2.78%
	146.82	<0.01%	<0.01%	<0.01%
	193.14	−2.22%	−2.22%	<0.01%

### Appendix A.2. High-Density Polyethylene HDPE Hiplex^®^ HHM5502 (HIP-Petrohemija, Pančevo, Serbia)

This section details the application of the pressure drop prediction methodology to High-density polyethylene HDPE Hiplex^®^ HHM5502 (HIP-Petrohemija, Pančevo, Serbia). Table A2 presents the experimental data collected.

**Table A2:** Experimental data for High-density polyethylene HDPE Hiplex^®^ HHM5502 (HIP-Petrohemija, Pančevo, Serbia) collected at varying operating conditions. $H_{3}$ is the die outlet gap [mm], $\dot{G}$ is the mass flow rate [kg/h], $p_{1}$ and $p_{2}$ are the pressures [MPa] at sensor locations one and two, $T_{1}$ and $T_{2}$ are the temperatures [°C] at sensor locations one and two, and $\Delta p_{EXP}$ is the pressure drop [MPa] calculated as $\left(p_{1}-p_{2}\right)$.

$H_{3}$ [mm]	$\dot{G}$ [kg/h]	$p_{1}$ [MPa]	$T_{1}$ [°C]	$p_{2}$ [MPa]	$T_{2}$ [°C]	$\Delta p_{EXP}$ [MPa]
3.0	37.26 ± 0.14	7.1 ± 0.5	196.9 ± 2.5	2.9 ± 0.5	205.0 ± 2.5	4.2 ± 0.7
3.0	82.86 ± 0.15	9.7 ± 0.5	197.0 ± 2.5	3.7 ± 0.5	205.5 ± 2.5	6.0 ± 0.7
3.0	130.2 ± 0.2	11.9 ± 0.5	197.2 ± 2.5	4.4 ± 0.5	206.2 ± 2.5	7.5 ± 0.7
3.0	178.32 ± 0.09	14.0 ± 0.5	197.2 ± 2.5	5.1 ± 0.5	206.8 ± 2.5	8.9 ± 0.7
3.0	240.00 ± 0.16	16.1 ± 0.5	197.1 ± 2.5	5.8 ± 0.5	208.0 ± 2.5	10.3 ± 0.7
4.5	37.14 ± 0.4	4.3 ± 0.5	200.2 ± 2.5	2.0 ± 0.5	201.4 ± 2.5	2.3 ± 0.7
4.5	83.0 ± 0.3	5.9 ± 0.5	200.2 ± 2.5	2.5 ± 0.5	201.5 ± 2.5	3.4 ± 0.7
4.5	131.0 ± 0.6	7.3 ± 0.5	200.4 ± 2.5	2.9 ± 0.5	202.1 ± 2.5	4.4 ± 0.7
4.5	180.36 ± 0.13	8.5 ± 0.5	200.3 ± 2.5	3.3 ± 0.5	202.4 ± 2.5	5.2 ± 0.7
4.5	238.9 ± 0.2	9.7 ± 0.5	200.2 ± 2.5	3.6 ± 0.5	202.9 ± 2.5	6.1 ± 0.7

Table A3 summarizes the self-consistency and cross-validation predictions. The results reinforce the main findings of this study: predictions are highly accurate when the similarity condition is met, and the error increases significantly when it is not, validating the condition as a reliable diagnostic tool.

**Table A3:** Relative error of pressure drop predictions for different Power Law model parameters at varying mass flow rates and die outlet gap $H_{3}$ for High-density polyethylene HDPE Hiplex^®^ HHM5502 (HIP-Petrohemija, Pančevo, Serbia). The table displays the relative error (% with sign) between the experimental pressure drop and the pressure drop predicted by the Power Law model for various combinations of $\bar{m}$ and $\bar{\phi }$. Results located on the main diagonal represent self-consistency predictions where the values of $\bar{m}$ and $\bar{\phi }$ used to calculate the pressure drop were derived from the same die. Conversely, values off the main diagonal represent cross-validated predictions, as the parameters were calculated using data from a different die. Values marked with an asterisk (*) indicate that the prediction does not meet the similarity condition and thus falls outside the model’s validated range of applicability. For the specific standard deviations associated with the experimental mass flow rates ($\dot{G}$), please refer to the primary data in Table A2.

In-Design Die $H_{3}$ [mm]	$\dot{G}$ [kg/h]	Relative Error (%)	
		Reference Die $H_{3}$ = 3.0 mm, $\bar{m}=2.1$ $\bar{\phi }=4.24\cdot 10^{-9}$ $\left[1/\left(Pa^{m}\cdot s\right)\right]$	Reference Die $H_{3}$ = 4.5 mm, $\bar{m}=1.9$ $\bar{\phi }=2.61\cdot 10^{-8}$ $\left[1/\left(Pa^{m}\cdot s\right)\right]$
3.0	37.26	<0.01%	<0.01%
	82.86	3.33%	5.00%
	130.2	2.67%	6.67%
	178.32	<0.01%	6.74% *
	240.00	<0.01%	7.77% *
4.5	37.14	4.35% *	<0.01%
	83.0	2.94%	2.94%
	131.0	<0.01%	2.27%
	180.36	−1.92%	1.92%
	238.9	−3.28%	<0.01%

### Appendix A.3. Polypropylene PP Moplen EP440G (LyondellBasell, Rotterdam, The Netherlands)

This section details the application of the pressure drop prediction methodology to Polypropylene PP Moplen EP440G (LyondellBasell, Rotterdam, The Netherlands). Table A4 presents the experimental data collected.

**Table A4:** Experimental data for Polypropylene PP Moplen EP440G (LyondellBasell, Rotterdam, The Netherlands) collected at varying operating conditions. $H_{3}$ is the die outlet gap [mm], $\dot{G}$ is the mass flow rate [kg/h], $p_{1}$ and $p_{2}$ are the pressures [MPa] at sensor locations one and two, $T_{1}$ and $T_{2}$ are the temperatures [°C] at sensor locations one and two, and $\Delta p_{EXP}$ is the pressure drop [MPa] calculated as $\left(p_{1}-p_{2}\right)$.

$H_{3}$ [mm]	$\dot{G}$ [kg/h]	$p_{1}$ [MPa]	$T_{1}$ [°C]	$p_{2}$ [MPa]	$T_{2}$ [°C]	$\Delta p_{EXP}$ [MPa]
1.5	31.38 ± 0.03	13.9 ± 0.5	191.4 ± 2.5	6.0 ± 0.5	206.0 ± 2.5	7.9 ± 0.7
1.5	67.98 ± 0.07	17.0 ± 0.5	191.4 ± 2.5	7.1 ± 0.5	206.0 ± 2.5	9.9 ± 0.7
1.5	106.26 ± 0.06	18.9 ± 0.5	191.5 ± 2.5	7.8 ± 0.5	207.1 ± 2.5	11.1 ± 0.7
1.5	142.62 ± 0.11	20.2 ± 0.5	191.7 ± 2.5	8.2 ± 0.5	208.9 ± 2.5	12.0 ± 0.7
1.5	187.56 ± 0.11	21.3 ± 0.5	191.9 ± 2.5	8.5 ± 0.5	210.7 ± 2.5	12.8 ± 0.7
3.0	31.44 ± 0.12	5.7 ± 0.5	196.8 ± 2.5	2.3 ± 0.5	205.2 ± 2.5	3.4 ± 0.7
3.0	69.06 ± 0.10	7.3 ± 0.5	196.9 ± 2.5	2.8 ± 0.5	205.3 ± 2.5	4.5 ± 0.7
3.0	106.3 ± 0.4	8.4 ± 0.5	197.2 ± 2.5	3.1 ± 0.5	205.7 ± 2.5	5.3 ± 0.7
3.0	144.72 ± 0.08	9.2 ± 0.5	197.2 ± 2.5	3.4 ± 0.5	206.0 ± 2.5	5.8 ± 0.7
3.0	188.10 ± 0.17	9.9 ± 0.5	197.4 ± 2.5	3.5 ± 0.5	206.6 ± 2.5	6.4 ± 0.7
4.5	32.88 ± 0.13	3.7 ± 0.5	200.3 ± 2.5	1.8 ± 0.5	201.5 ± 2.5	1.9 ± 0.7
4.5	69.48 ± 0.04	4.7 ± 0.5	200.2 ± 2.5	2.1 ± 0.5	201.4 ± 2.5	2.6 ± 0.7
4.5	106.98 ± 0.13	5.4 ± 0.5	200.3 ± 2.5	2.3 ± 0.5	201.6 ± 2.5	3.1 ± 0.7
4.5	144.36 ± 0.18	6.0 ± 0.5	200.4 ± 2.5	2.4 ± 0.5	201.7 ± 2.5	3.6 ± 0.7
4.5	189.48 ± 0.19	6.5 ± 0.5	200.6 ± 2.5	2.6 ± 0.5	202.1 ± 2.5	3.9 ± 0.7

Table A5 summarizes the self-consistency and cross-validation predictions. The results reinforce the main findings of this study: predictions are highly accurate when the similarity condition is met, and the error increases significantly when it is not, validating the condition as a reliable diagnostic tool.

**Table A5:** Relative error of pressure drop predictions for different Power Law model parameters at varying mass flow rates and die outlet gap $H_{3}$ for Polypropylene PP Moplen EP440G (LyondellBasell, Rotterdam, The Netherlands). The table displays the relative error (% with sign) between the experimental pressure drop and the pressure drop predicted by the Power Law model for various combinations of $\bar{m}$ and $\bar{\phi }$. Results located on the main diagonal represent self-consistency predictions where the values of $\bar{m}$ and $\bar{\phi }$ used to calculate the pressure drop were derived from the same die. Conversely, values off the main diagonal represent cross-validated predictions, as the parameters were calculated using data from a different die. Values marked with an asterisk (*) indicate that the prediction does not meet the similarity condition and thus falls outside the model’s validated range of applicability. For the specific standard deviations associated with the experimental mass flow rates ($\dot{G}$), please refer to the primary data in Table A4.

In-Design Die $H_{3}$ [mm]	$\dot{G}$ [kg/h]	Relative Error (%)		
		Reference Die $H_{3}$ = 1.5 mm, $\bar{m}=3.7$ $\bar{\phi }=1.57\cdot 10^{-16}$ $\left[1/\left(Pa^{m}\cdot s\right)\right]$	Reference Die $H_{3}$ = 3.0 mm, $\bar{m}=2.8$ $\bar{\phi }=2.19\cdot 10^{-12}$ $\left[1/\left(Pa^{m}\cdot s\right)\right]$	Reference Die $H_{3}$ = 4.5 mm, $\bar{m}=2.4$ $\bar{\phi }=1.70\cdot 10^{-10}$ $\left[1/\left(Pa^{m}\cdot s\right)\right]$
1.5	31.38	<0.01%	2.53%	1.27%
	67.98	−2.02%	7.07%	11.11% *
	106.26	−0.90%	11.71% *	18.92% *
	142.62	−0.83%	15.00% *	24.17% *
	187.56	<0.01%	18.75% *	29.69% *
3.0	31.44	11.76% *	<0.01%	−8.82%
	69.06	4.44% *	<0.01%	−4.44%
	106.3	−1.89%	−1.89%	−3.77%
	144.72	−1.72%	<0.01%	<0.01% *
	188.10	−4.69%	<0.01%	<0.01% *
4.5	32.88	36.84% *	15.79% *	<0.01%
	69.48	23.08% *	11.54%	<0.01%
	106.98	12.90% *	6.45%	<0.01%
	144.36	5.56%	2.78%	−2.78%
	189.48	5.13%	5.13%	<0.01%

## Appendix B

### Appendix B.1. Metrological Framework and Process Variance

To ensure the statistical rigor of the experimental data presented in the main text and to address measurement reproducibility, a comprehensive uncertainty budget was developed. The fluctuations in operating parameters and the physical limitations of the industrial instrumentation were analyzed by combining two primary components:

1. Process variance ($\sigma_{process}$): Calculated as the sample standard deviation to capture natural process fluctuations under steady-state conditions. For pressure and temperature, it is derived from continuous time-series data recorded at a 1 Hz sampling rate over a 60 s window (n=60). For mass flow rate, it is calculated from the weights of three independent physical samples, each collected over a 60 s extrusion interval (n=3).
2. Instrument uncertainty ($\epsilon_{instrument}$): Determined based on the technical specifications provided by the sensor manufacturers and the tolerances defined by international reference standards.

The combined standard uncertainty ($u_{c}$) for each directly measured variable was calculated using the root sum square (RSS) method,

(A1) $$u_{c}=\sqrt{\sigma_{process}^{2}+\epsilon_{instrument}^{2}}$$

### Appendix B.2. Application to Primary Measurements

The variance analysis revealed distinct behaviors depending on the measured variable. For the mass flow rate ($\dot{G}$), the intrinsic precision of the analytical balance (0.01 g) is experimentally negligible. The primary source of error is the human reaction time involved in manual sampling (estimated in 0.5 s). However, by collecting three independent physical replicates over 60 s intervals, this timing error and the natural volumetric fluctuations of the extruder are intrinsically captured by the sample standard deviation (σ). Therefore, process variance dictates the mass flow rate uncertainty.

Conversely, for pressure ($P_{Melt}$, $P_{1}$ and $P_{2}$) and temperature ($T_{1}$ and $T_{2}$) measurements, sampling analysis showed that the physical process fluctuations are significantly smaller than the baseline accuracy of the industrial sensors. Consequently, the fixed instrument uncertainty overwhelmingly dominates the combined uncertainty for these parameters. See Table A6 for a practical example.

**Table A6:** Detailed metrological analysis for the operating condition $\dot{G}=31.50\;kg/h$ and outlet gap $H_{3}=1.5\;mm$ for the material Borealis BB125MO. The budget clearly illustrates the dominant factors driving the final rounded uncertainties reported in the primary data tables.

Source of Uncertainty/Parameter	$\dot{G}$ [kg/h]	$p_{melt}$ [MPa]	$p_{1}$ [MPa]	$T_{1}$ [°C]	$p_{2}$ [MPa]	$T_{2}$ [°C]
Mean value (μ)	31.50	19.9	14.8	196.0	6.4	206.1
Process variance (σ)	0.03	0.06	0.06	0.08	0.05	0.3
Instrument uncertainty (ϵ)	Negligible *	0.5	0.5	2.5	0.5	2.5
Combined uncertainty ($u_{c}$)	0.03	0.50	0.50	2.50	0.50	2.52
Dominant error factor	Process Variance	Instrument Uncertainty	Instrument Uncertainty	Instrument Uncertainty	Instrument Uncertainty	Instrument Uncertainty
Rounded value, reported in main text	0.03	0.5	0.5	2.5	0.5	2.5

* Note: The 0.01 g intrinsic error of the analytical balance is negligible. The timing error from manual sampling is inherently captured within the standard deviation of the three physical replicates.

### Appendix B.3. Error Propagation for Pressure Drop ($∆p_{EXP}$)

Because the experimental pressure drop ($∆p_{EXP}$) is not directly measured but derived from the algebraic difference between two independent sensor signals along the die, the total propagated uncertainty ($u_{∆p}$) was determined using the standard error propagation law for linear combinations of independent variables:

(A2) $$u_{∆p}=\sqrt{u_{p1}^{2}+u_{p2}^{2}}$$

Given the established dominant instrument uncertainty of 0.5 MPa for each individual pressure sensor, the propagation calculation yields:

(A3) $$u_{∆p}=\sqrt{0.5^{2}+0.5^{2}}=0.7\;MPa$$

This calculated value of 0.7 MPa represents the absolute, conservative error bound applied systematically to all experimental pressure drop evaluations in this study.
